# Maternal Plane of Nutrition during Late Gestation and Weaning Age Alter Angus × Simmental Offspring Longissimus Muscle Transcriptome and Intramuscular Fat

**DOI:** 10.1371/journal.pone.0131478

**Published:** 2015-07-08

**Authors:** Sonia J. Moisá, Daniel W. Shike, Lindsay Shoup, Sandra L. Rodriguez-Zas, Juan J. Loor

**Affiliations:** 1 Mammalian NutriPhysioGenomics, Department of Animal Sciences, University of Illinois, Urbana, Illinois, United States of America; 2 Department of Animal Sciences, University of Illinois, Urbana, Illinois, United States of America; 3 The Institute for Genomic Biology, University of Illinois, Urbana, Illinois, United States of America; 4 Division of Nutritional Sciences, Illinois Informatics Institute, University of Illinois, Urbana, Illinois, United States of America; INRA, FRANCE

## Abstract

In model organisms both the nutrition of the mother and the young offspring could induce long-lasting transcriptional changes in tissues. In livestock, such changes could have important roles in determining nutrient use and meat quality. The main objective was to evaluate if plane of maternal nutrition during late-gestation and weaning age alter the offspring’s *Longissimus* muscle (LM) transcriptome, animal performance, and metabolic hormones. Whole-transcriptome microarray analysis was performed on LM samples of early (EW) and normal weaned (NW) Angus × Simmental calves born to grazing cows receiving no supplement [low plane of nutrition (LPN)] or 2.3 kg high-grain mix/day [medium plane of nutrition (MPN)] during the last 105 days of gestation. Biopsies of LM were harvested at 78 (EW), 187 (NW) and 354 (before slaughter) days of age. Despite greater feed intake in MPN offspring, blood insulin was greater in LPN offspring. Carcass intramuscular fat content was greater in EW offspring. Bioinformatics analysis of the transcriptome highlighted a modest overall response to maternal plane of nutrition, resulting in only 35 differentially expressed genes (DEG). However, weaning age and a high-grain diet (EW) strongly impacted the transcriptome (DEG = 167), especially causing a lipogenic program activation. In addition, between 78 and 187 days of age, EW steers had an activation of the innate immune system due presumably to macrophage infiltration of intramuscular fat. Between 187 and 354 days of age (the “finishing” phase), NW steers had an activation of the lipogenic transcriptome machinery, while EW steers had a clear inhibition through the epigenetic control of histone acetylases. Results underscored the need to conduct further studies to understand better the functional outcome of transcriptome changes induced in the offspring by pre- and post-natal nutrition. Additional knowledge on molecular and functional outcomes would help produce more efficient beef cattle.

## Introduction

The prenatal periods during which the organism is susceptible to environmental stimuli leading to fetal programming are the embryonic phase, the mid-gestation period (organogenesis), and late gestation (rapid growth). The effects of maternal nutrition on fetal growth and its carry-over effects on offspring growth and development were reviewed recently. As an example, extreme intrauterine growth retardation can result in slower growth throughout postnatal life [1]. Maternal nutrition seems to elicit different outcomes in the offspring depending on the gestational stage during which treatments begin. For example, protein supplementation of grazing cows during late gestation in the winter enhanced feedlot performance and carcass quality of the offspring.

In a review on fetal programming and skeletal muscle development in the ruminant [2], it was concluded that adipogenesis is initiated during mid-gestation. At this time, a pool of undifferentiated mesenchymal stem cells is present. From this pool, either myocytes or adipocytes are able to differentiate from committed mesenchymal cells to become skeletal muscle or adipose tissue [3]. The increase in number of stem cells throughout middle-to-late gestation led to the hypothesis that nutritional management has the potential to be more effective during the prenatal period rather than the postnatal portion of an animal’s life [2]. In sheep, a study revealed that differences in maternal nutrition during mid-to-late gestation can impact the programing of fetal muscle and fat tissues [4]. However, it remains to be determined if plane of nutrition during late-pregnancy (last 90 days) can elicit carryover effects acquired through programming in beef cattle.

There are several maternal-nutrition studies utilizing real-time RT-PCR to evaluate specific target genes in adipose tissue or longissimus muscle (LM) of beef [5], lamb [6] and sheep [7] offspring. Despite these efforts, to the best of our knowledge, there are no published studies of whole-transcriptome profiles in LM of offspring from mothers fed high or low planes of nutrition during late-pregnancy in beef cattle.

Our hypothesis was that a high-plane of nutrition of the cow during late gestation would result in early activation of genes associated with myogenesis, adipogenesis, lipogenesis and the synthesis of adipokines in the offspring’s skeletal muscle. Furthermore, changes in nutrition of the pregnant mother also would elicit alterations associated with epigenetic regulation of gene expression. The objectives of this study were to assess the effect of maternal plane of nutrition and early weaning to a high-grain diet on the skeletal muscle transcriptome of the offspring.

## Materials and Methods

Animal use in this study was approved by the Institutional Animal Care and Use Committee (**IACUC**) of the University of Illinois. A subset of 20 Angus × Simmental beef cows from the University of Illinois Dixon Springs Agriculture Center in Simpson, IL (USA), were selected from a group of animals utilized in a parallel study [8]. Main effects evaluated were maternal plane of nutrition during late gestation and postnatal management of the offspring. Three months prior to the projected parturition date cows were assigned to treatments (low or medium plane of nutrition) in a split-plot design. Low plane of nutrition (**LPN**) was achieved by grazing endophyte-infected tall fescue/red clover pastures during July, August, and September with no supplement. Medium plane of nutrition cow diet (**MPN**) was achieved by grazing endophyte-infected tall fescue/red clover pastures supplemented daily with 2.3 kg of dried distiller’s grains with solubles and soyhulls (70% DDGS/30% soyhulls). Cow supplementation was initiated at 103 ± 11 days prepartum while on pasture and it was halted at the midpoint of parturition (2 ± 11 days postpartum). More information about cow supplementation is reported elsewhere [8].

Composition of the diet [dry matter (**DM**) basis] fed to early weaned (**EW**) steers upon arrival to the feedlot and prior to normal weaning (**NW**), and the feedlot diet fed to EW and NW steers after normal weaning are reported in Table 1. Angus × Simmental steer calves were randomly assigned to early or normal weaning (EW or NW) treatments within each gestational treatment. At 78 ± 2 days postpartum, EW offspring were weaned, transported to University of Illinois Beef and Sheep Field Laboratory (Urbana, IL, USA), and adapted to a high-grain diet until they had *ad libitum* consumption. At 187 ± 2 days postpartum, NW offspring were weaned and transported to University of Illinois Beef and Sheep Field Laboratory. All offspring were co-mingled among treatments.

**Table 1:** Composition of diet (DM basis) fed to early wean (EW) steers upon arrival to feedlot and prior to normal weaning^1^ and feedlot diet fed to EW and normal wean (NW) steers after normal weaning.

	Inclusion, % DM	
Item	EW diet	Feedlot diet
Ingredient, %		
MWDGS^2^	45	45
Dry Whole Corn	25	25
High Moisture Corn	—	—
Corn Husklage	20	20
Ground corn	7.3	7.3
Limestone	2.5	2.5
Trace mineral salt^3^	0.1	0.1
Rumensin 90^4^	0.018	0.018
Tylosin 40^5^	0.012	0.012
Soybean oil	0.076	0.076
Analyzed nutrient content, %		
Crude protein	17.3	18.1
Neutral detergent fiber	23.9	25.3
Acid detergent fiber	14.1	14.3
Crude fat	5.3	5.1

^1^Age at weaning: EW = 78 ± 11 days of age; NW = 186 ± 11 days of age.

^2^MWDGS = Modified Wet Distillers Grains with Solubles.

^3^Trace Mineral Salt = 8.5% Ca (as CaCO3), 5% Mg (as MgO and MgSO4), 7.6% K (as KCl2), 6.7% Cl (as KCl2) 10% S (as S8, prilled), 0.5% Cu (as CuSO4 and Availa-4 (Zinpro Performance Minerals; Zinpro Corp, Eden Prairie, MN)), 2% Fe (as FeSO4), 3% Mn (as MnSO4 and Availa-4), 3% Zn (as ZnSO4 and Availa-4), 278 ppm Co (as Availa-4), 250 ppm I (as Ca(IO3)2), 150 Se (Na2SeO3), 2,205 KIU/kg Vit A(as retinyl acetate), 662.5 KIU/kg Vit D (as cholecalciferol), 22,047.5 IU/kg Vit E (as DL-α-tocopheryl acetate), and less than 1% CP, fat, crude fiber, salt.

^4^Rumensin 90 (198 g monensin/kg Rumensin 90; Elanco Animal Health, Greenfield, IN, USA).

^5^Tylosin 40 (88 g tylan/kg Tylosin 40; Elanco Animal Health, Greenfield, IN, USA).

Blood was collected from the jugular vein at 78, 187 and 296 days of age to isolate serum for insulin (Bovine Insulin ELISA kit, Cat No. 10–1201–01, Mercodia AB, Uppsala, Sweden), glucose (Hexokinase G-6-PDH method using a Beckman Coulter, Fullerton, CA, USA; Diagnostics Laboratory, College of Veterinary Medicine, University of Illinois, Urbana, USA) and adiponectin (Millipore, LA, USA). The latter was determined using a liquid RIA (Millipore, LA, USA) following a protocol previously described [9]. After normal weaning, all offspring were placed on a common, grain-based finishing diet that is typical of industry management [crude protein (**CP**) %, 18.1, neutral detergent fiber (**NDF**) %, 25.3, acid detergent fiber (**ADF**) %, 14.3, crude fat %, 5.1] (Table 1). All the offspring in the study were harvested at a commercial packing plant when they reached the selected end point target back fat thickness of 1.1 cm. Reported final body weight (**BW**) was calculated from hot carcass weight using a 62% dressing percentage.

LM biopsies were harvested from a subset of 5 animals per gestational × postnatal treatment from the main herd at ~78 days of age, ~187 days, and during the last week prior to harvest (~354 days). Selection of steer progeny for biopsy was performed based on 2 criteria: first, offspring for biopsy were selected based on their dam’s performance. Only offspring from cows whose BW and BW change during late gestation was within ½ of a standard deviation on either side of the mean (LPN or MPN) were considered for biopsy. Selecting based on cow BW and BW change ensured that only calves from cows that were representative of their treatment were utilized for transcriptomics. The final selection of steers for biopsy was based on steer BW. Only steers whose BW was within ½ of a standard deviation on either side of the mean were utilized. This selection criterion strategy minimized the effects of variation in dam’s milk production, which was not significantly different between LPN and MPN treatments [8].

Transcriptomics was performed with a transcriptome-wide bovine microarray (Agilent-015354 Bovine Oligo Microarray-4x44K) that contains 21,475 unique genes and transcripts of Bos Taurus, with two probes per gene. The methods used for hybridization and scanning were according to manufacturer’s protocols and Loor *et al* [10]. The microarray data were deposited in the National Center for Biotechnology Information (**NCBI**) Gene Expression Omnibus (**GEO**) database (http://www.ncbi.nlm.nih.gov/gds) with accession number GSE65560.

### Data mining

The entire microarray data set with associated statistical *P*-values were imported into Ingenuity Pathways Analysis (**IPA**, www.ingenuity.com) in order to examine the number of activated and inhibited differentially expressed genes (**DEG**). Entrez Gene IDs were used to identify individual sequences.

### Statistical analysis

Data from the microarray analysis were normalized for dye and microarray effects (i.e., Lowess normalization and array centering) and used for statistical analysis. The MIXED procedure of SAS (SAS Institute, Inc., Cary, NC, USA) was used for statistical analysis. Fixed effects were treatment (EW, NW), diet (LPN, MPN), time (78, 187, and 354 days of age), first, second and third order interactions between diet, time and treatment, and dye (Cy3, Cy5) and random effects included steer and microarray. Raw *P* values were adjusted using Benjamini and Hochberg’s false discovery rate (**FDR**).

The statistical model used was: Yijklm = μ + T_i_ + D_j_ + W_k_ +S_l_ + (T × D)_ij_ + (D × W)_jk_ + (T × W)_ik_ + (D × T × W)_ijk_ + ε_ijklm_; where, Y_ijklm_ is the background-adjusted normalized fold change value; μ is the overall mean; T_i_ is the fixed effect of time (3 levels); D_j_ is the fixed effect of cow plane of nutrition (2 levels); W_k_ is the fixed effect of weaning age (2 levels); S_l_ is the random effect of steer nested within treatment; T × D, D × W, T × W are the interactions of time by diet, diet by wean and time by wean, respectively; D × T × W is the interaction of third order for the main effects; and ε_ijklm_ is the random error (0, σ_e_^2^) associated with Y_ijklm_. All means were compared using the PDIFF statement of SAS (SAS Institute, Inc., Cary, NC, USA). Statistical significance was declared at *P* ≤ 0.05 and FDR ≤ 0.10. Animal feedlot performance, carcass quality parameters, ultrasound and blood data were also analyzed using the MIXED procedure of SAS, and treatment was the fixed effect in the statistical model. The random effect in all models was steer within treatment.

### Dynamic impact approach (DIA)

Bioinformatics analysis of microarray data was performed using DIA [11] and information from the freely-available online databases Kyoto Encyclopedia of Genes and Genomes (**KEGG**) and Database for Annotation, Visualization, and Integrated Discovery (**DAVID**) v6.7 databases. A list of gene identifiers (Entrez Gene IDs) was uploaded all at once to extract and summarize functional annotations associated with groups of genes or with each individual gene. The significance value associated with biological processes and pathways is a measure of the likelihood that the distribution of DEG in these pathways and biological processes is due to chance. The significance is expressed as a *P*-value, which is calculated using the right-tailed Fisher's Exact Test and adjusted using FDR. Details of the DIA approach and its validation have been reported previously [11]. The interpretation of the bioinformatics analysis was performed following the same approach as our previous study [12].

## Results

### Animal performance

Performance data for the entire group of animals on study are reported elsewhere [8]. Feedlot performance of EW and NW steers used for transcriptomics is presented in Table 2. Only initial BW at the time steers entered the feedlot was significant (*P* < 0.01) with EW steers being heavier as compared with NW steers. Dry matter intake (**DMI**) was higher for MPN as compared with LPN steers (*P* = 0.03). Ultrasound at the time of EW (78 days of age) revealed no significant differences (*P* > 0.05) between treatments (i.e. all possible combinations between weaning age and cow plane of nutrition) for marbling and back fat thickness (Table 2).

**Table 2:** Feedlot performance, ultrasound measurements and carcass quality parameters for Angus × Simmental steers (n = 20) from cows that received a low (LPN) or a medium (MPN) plane of nutrition (D) during the late gestation period. Weaning times (W) are early weaning (EW) and normal weaning (NW).

	Treatments							
	EW		NW			*P*-value^1^		
Item	LPN	MPN	LPN	MPN	SEM	D	W	D*W
Feedlot Performance								
Initial BW	258	278	212	204	18.25	0.73	<0.01	0.42
Final BW^2^	548	581	524	533	19.78	0.26	0.07	0.52
ADG (kg/d)	1.74	1.66	1.81	1.69	0.15	0.40	0.56	0.85
DMI (kg/d)	7.84	9.33	8.18	8.48	0.41	0.03	0.52	0.14
Ultrasound measurements								
BF at EW (cm)	0.31	0.35	0.33	0.34	0.02	0.13	0.71	0.52
BF at NW (cm)	0.33	0.33	0.31	0.33	0.02	0.54	0.57	0.74
Marbling at EW	427	388	434	419	27.80	0.30	0.48	0.65
Marbling at NW	330	409	376	418	40.41	0.13	0.47	0.63
Carcass quality parameters								
HCW (kg)	340	360	325	331	12.27	0.27	0.07	0.51
Calculated YG	3.0	3.0	2.5	2.3	0.27	1.00	0.03	1.00
LM area (cm^2^)	77.3	82.5	78.6	79.4	3.27	0.34	0.76	0.48
Marbling	418	573	480	482	43.13	0.07	0.72	0.07
Back fat thickness (cm)	1.24	1.37	1.30	1.12	0.13	0.83	0.41	0.22
KPH (%)	2.1	2.1	2.3	2.1	0.12	0.29	0.28	0.70
Days to harvest	367	377	392	412	11.10	0.16	0.01	0.59

^1^D = cow plane of nutrition effect (diet), W = weaning age effect, D × W = diet × weaning interaction.

^2^Based on 62% dressing percentage.

Hot carcass weight did not differ (*P* > 0.10) (Table 2). Calculated yield grade had a weaning effect (*P* = 0.03) with lower values for NW as compared with EW steers. Marbling score was greater (*P* = 0.07) for EW-MPN steers. Lastly, there was a weaning effect (*P* = 0.01) associated with EW steers reaching the back-fat thickness target end-point earlier than NW steers (Table 2).

**Fig 1:**
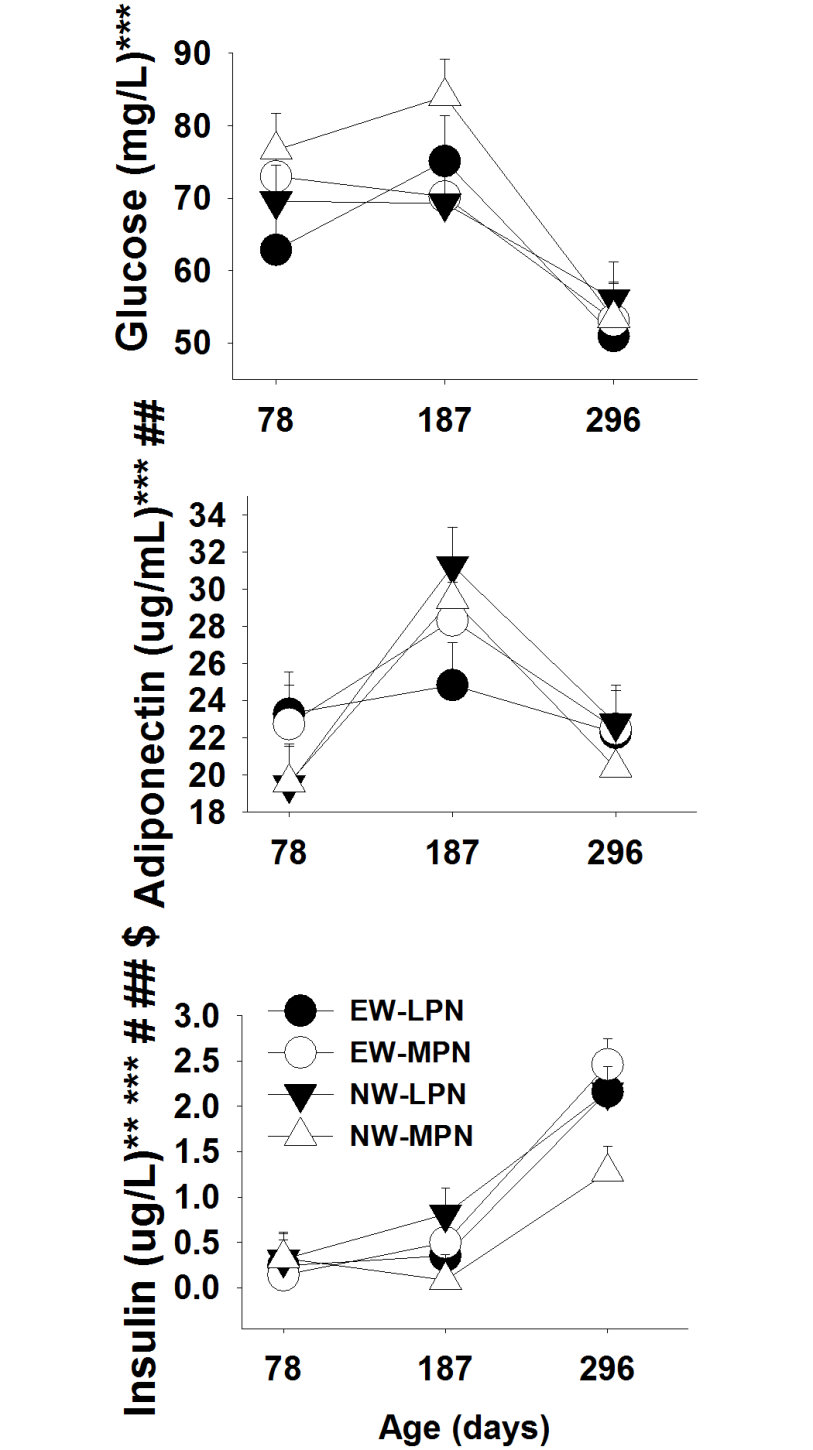
Glucose, adiponectin and insulin serum concentrations in Angus × Simmental steers from cows that received a low (LPN) or medium (MPN) plane of nutrition during the late gestation period. Weaning times are early wean (EW) and normal wean (NW). * Weaning, ** Diet, *** Time, # weaning × diet, ## weaning × time, ### diet × time and $ time × weaning × diet interaction effects (*P* < 0.05).

Serum glucose concentration had a significant time effect (*P* < 0.05) with lower values at 296 days of age (Fig 1). Adiponectin concentration had a significant cow plane of nutrition (diet), time, and diet × time interaction (*P* < 0.05) with increasing concentrations between 78 and 187 days of age and a switch to decreasing concentrations after 187 days of age for NW steers, but serum adiponectin did not change for EW steers (Fig 1). Insulin concentration was affected by weaning age, time, and weaning × time (*P* < 0.05) with a relatively constant concentration between 78 and 187 days of age for all treatments and a noticeable increase in concentration between 187 and 296 days of age (Fig 1).

### Microarray analysis

**Fig 2:**
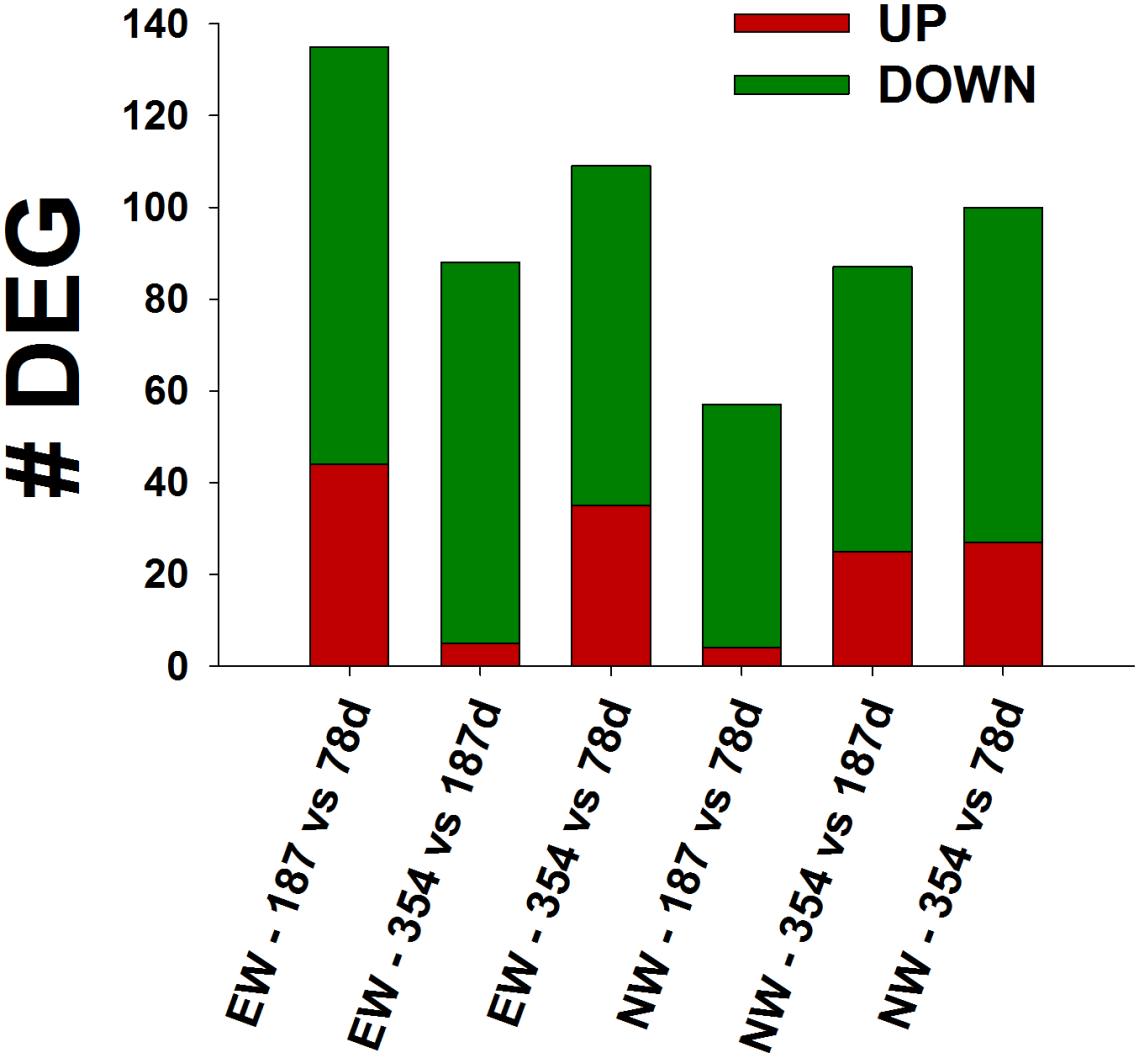
Differentially expressed genes (DEG; FDR < 0.10 and uncorrected *P* value < 0.05) in early wean (EW) and normal wean (NW) steers during the growing (78 to 187 days of age), finishing (187 to 354 days of age) and growing and finishing phases (78 to 354 days of age). Number of DEG for each comparison are those with overall FDR < 0.10, uncorrected *P* value < 0.05, and *P* < 0.05 between the specific time comparisons.

At an FDR < 0.10 (uncorrected *P* value < 0.05), a total of 35 DEG were affected overall by the cow plane of nutrition (Table 3), 145 DEG were affected by weaning time and 7,639 DEG were affected by time. In addition, there were 13 DEG for the time × wean × diet interaction (Table 4), 43 DEG for the time × diet, 31 DEG for the wean × diet interaction, and 167 DEG for the wean × time interaction. The 167 DEG for the wean × time interaction were analyzed separately for EW and NW steers: between 78 and 187 days of age, 187 and 354 days of age and between 87 and 354 days of age. For this analysis we utilized a cutoff *P* value < 0.05 for any given comparison (Fig 2).

**Table 3:** Symbol, entrez gene ID, log ratio expression value [low plane of nutrition (LPN) vs. medium plane of nutrition (MPN], type of molecule and localization in the cell for the 35 differentially expressed genes affected by cow plane of nutrition.

Symbol	Entrez Gene Name	Log Ratio	p-value	Location^1^	Type(s)^2^
*ABHD11*	abhydrolase domain containing 11	0.30	8.74E-06	Cytoplasm	enzyme
*ACLY*	ATP citrate lyase	0.35	7.74E-05	Cytoplasm	enzyme
*AGPAT6*	1-acylglycerol-3-phosphate O-acyltransferase 6	-0.22	5.90E-05	Cytoplasm	enzyme
*ARAP1*	ArfGAP with RhoGAP domain, ankyrin repeat and PH domain 1	-0.44	1.53E-04	Cytoplasm	other
*ART3*	ADP-ribosyltransferase 3	-0.78	4.64E-05	Plasma Memb.	enzyme
*ATP5S*	ATP synthase, H+ transporting, mitochondrial Fo complex, subunit s	0.33	1.09E-04	Cytoplasm	transporter
*C15orf40*	chromosome 15 open reading frame 40	-0.21	1.72E-04	Other	other
*C8orf48*	chromosome 8 open reading frame 48	-0.72	2.18E-05	Other	other
*CARD14*	caspase recruitment domain family, member 14	0.96	4.31E-05	Cytoplasm	other
*CHST12*	carbohydrate (chondroitin 4) sulfotransferase 12	0.78	5.32E-05	Cytoplasm	enzyme
*DEXI*	Dexi homolog (mouse)	0.29	1.50E-04	Other	other
*DHDH*	dihydrodiol dehydrogenase (dimeric)	0.40	8.49E-08	Other	enzyme
*DYNLL1*	dynein, light chain, LC8-type 1	0.47	2.29E-05	Cytoplasm	other
*EDC3*	enhancer of mRNA decapping 3	0.55	2.31E-05	Cytoplasm	other
*EHD4*	EH-domain containing 4	0.60	1.39E-04	Plasma Memb.	enzyme
*ENTPD2*	ectonucleoside triphosphate diphosphohydrolase 2	0.53	7.24E-05	Cytoplasm	enzyme
*EPC1*	enhancer of polycomb homolog 1 (Drosophila)	-0.28	9.13E-05	Nucleus	TR
*FCAR*	Fc fragment of IgA, receptor for	-0.52	6.40E-05	Plasma Memb.	other
*GSTA4*	glutathione S-transferase, alpha 4	-1.74	1.37E-05	Other	enzyme
*HLF*	hepatic leukemia factor	0.72	1.69E-04	Nucleus	TR
*IQGAP1*	IQ motif containing GTPase activating protein 1	-0.55	5.28E-05	Cytoplasm	other
*KRT74*	keratin 74	-1.01	7.04E-05	Cytoplasm	other
*LOC789391*	tRNA methyltransferase catalytic subunit TRMT61A like	-0.76	1.07E-04	Nucleus	enzyme
*MARCH7*	membrane-associated ring finger 7, E3 ubiquitin protein ligase	-0.62	7.57E-09	Extracell. Space	other
*MSRB1*	methionine sulfoxide reductase B1	-0.49	3.86E-05	Other	other
*NR2C2*	nuclear receptor subfamily 2, group C, member 2	-0.27	7.21E-05	Nucleus	LDNR
*NSMCE4A*	non-SMC element 4 homolog A (S. cerevisiae)	0.34	1.67E-04	Nucleus	other
*PNMAL1*	paraneoplastic Ma antigen family-like 1	0.89	1.31E-04	Other	other
*PSPH*	phosphoserine phosphatase	0.74	1.62E-04	Cytoplasm	phosphatase
*PYCR1*	pyrroline-5-carboxylate reductase 1	0.47	8.97E-05	Cytoplasm	enzyme
*SCUBE1*	signal peptide, CUB domain, EGF-like 1	1.05	1.03E-04	Plasma Memb.	transm. receptor
*SERF1A*	small EDRK-rich factor 1A (telomeric)	0.41	1.48E-04	Other	other
*SRSF5*	serine/arginine-rich splicing factor 5	-0.53	9.98E-05	Nucleus	other
*TAGLN3*	transgelin 3	0.34	1.17E-04	Extracell Space	other
*TOMM34*	translocase of outer mitochondrial membrane 34	0.36	1.14E-04	Cytoplasm	other

^1^Extracell. Space = extracellular space; Plasma Memb. = plasma membrane; G receptor = G protein coupled receptor.

^2^TR = transcription regulator; LDNR—ligand-dependent nuclear receptor; Transm. Receptor = transmembrane receptor.

**Table 4:** Symbol, entrez gene ID, log ratio expression value, type of molecule and localization in the cell for the 13 differentially expressed genes affected by the weaning × diet × time interaction.

Symbol	Entrez Gene Name	Log Ratio	p-value	Location^1^	Type(s)^2^
*ARID1A*	AT rich interactive domain 1A (SWI-like)	2.885	2.04E-04	Nucleus	TR
*BTG1*	B-cell translocation gene 1, anti-proliferative	-1.262	2.07E-03	Nucleus	TR
*EPAS1*	endothelial PAS domain protein 1	-1.797	2.08E-01	Nucleus	TR
*HSBP1*	heat shock factor binding protein 1	1.064	2.38E-02	Nucleus	TR
*IMPG1*	interphotoreceptor matrix proteoglycan 1	1.577	2.22E-05	Extracell. Space	other
*KIAA0232*	KIAA0232	1.889	3.33E-04	Extracell. Space	other
*KRTDAP*	keratinocyte differentiation-associated protein	-12.672	8.49E-01	Extracell. Space	other
*MARK4*	MAP/microtubule affinity-regulating kinase 4	-1.247	2.99E-02	Cytoplasm	kinase
*NME1*	NME/NM23 nucleoside diphosphate kinase 1	-5.373	1.17E-01	Cytoplasm	kinase
*OR51F1*	olfactory receptor, family 51, subfamily F, member 1	-1.393	4.66E-04	Plasma Memb.	G receptor
*OTX1*	orthodenticle homeobox 1	-3.683	2.31E-01	Nucleus	TR
*PLAGL1*	pleiomorphic adenoma gene-like 1	1.313	5.06E-03	Nucleus	TR
*SLC25A5*	solute carrier family 25, member 5	-4.401	2.36E-01	Cytoplasm	transporter

^1^Extracell. Space = extracellular space; Plasma Memb. = plasma membrane.

^2^TR = trascription regulator; G receptor = G protein coupled receptor.

When focusing on the 167 DEG due to time × treatment interaction, the highest number of DEG, 91 down-regulated and 44 up regulated (uncorrected *P* value < 0.05; FDR < 0.10), were detected in EW steers between 78 and 187 days of age (Fig 2). During the same time-frame, NW steers had the lowest DEG with 53 downregulated and 4 upregulated genes (uncorrected *P* value < 0.05; FDR < 0.10). It is noteworthy that the number of DEG in NW steers increased markedly between 187 and 354 days of age. Thus, when comparing the responses between 78 and 354 day of age, EW and NW steers had a similar number of DEG (Fig 2).

**Fig 3:**
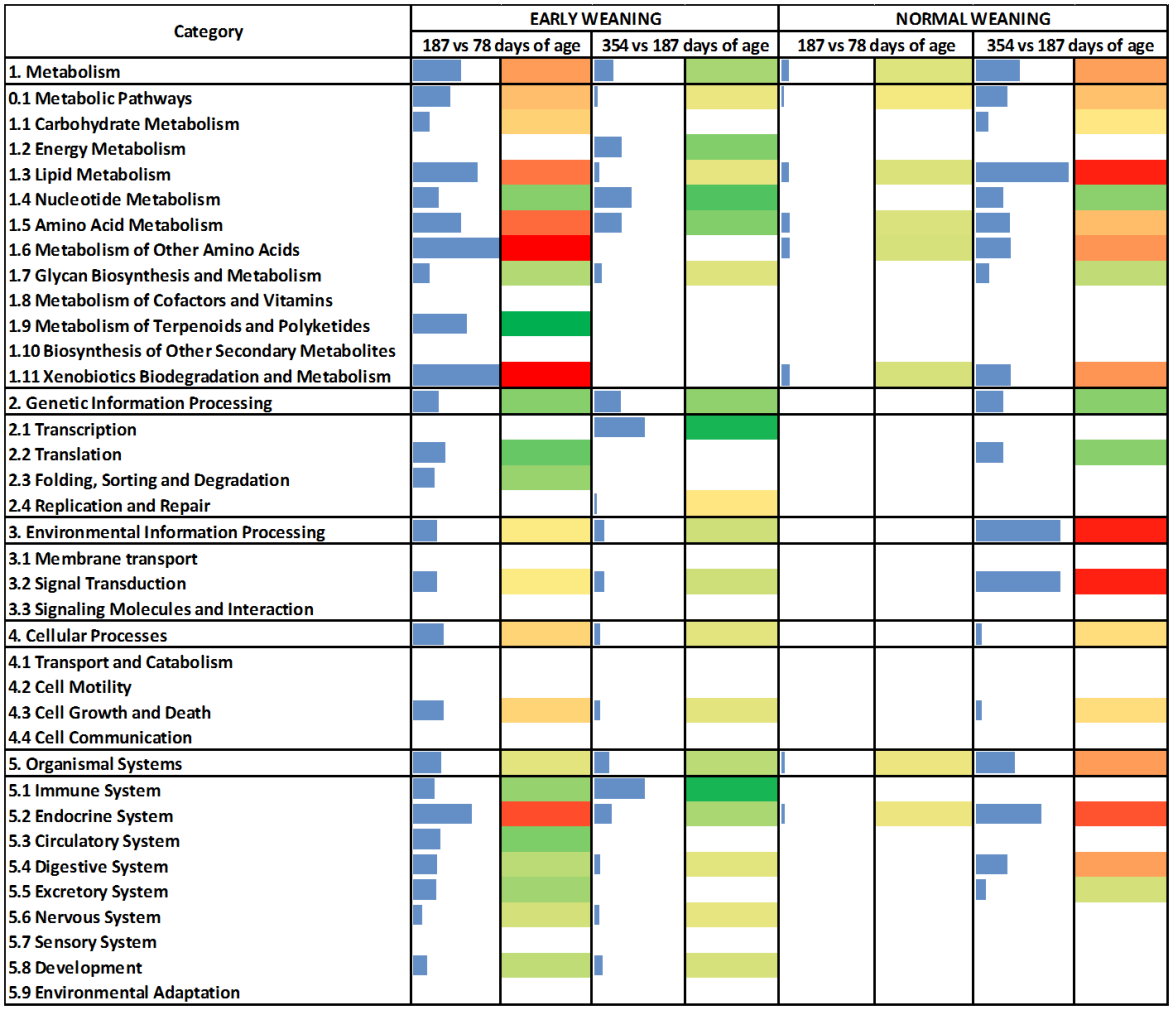
Dynamic Impact Approach (DIA) of differentially expressed genes (P value < 0.05; FDR < 0.10) on the Kyoto Encyclopedia of Genes and Genomes (KEGG) Pathways database. Flux represents the direction of each category and the corresponding subcategory (green color = inhibition, yellow color = stable, red color = activation with different color intensities according with the level of up-regulation or down-regulation). Blue bars denote the impact of each category and the corresponding subcategories.

**Fig 4:**
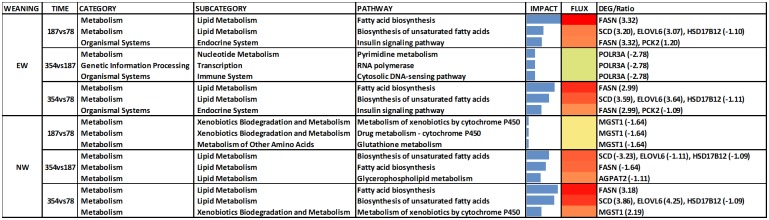
Results of the 3 most impacted pathways within differentially expressed genes affected during the growing phase (187 vs. 78 d), finishing phase (354 vs. 187 d) and the whole experiment (354 vs. 78 d). Analysis performed using the Dynamic Impact Approach (DIA) based on the Kyoto Encyclopedia of Genes and Genomes (KEGG) Pathways database. Flux represents the direction of each category and the corresponding subcategory (green color = inhibition, yellow color = stable, red color = activation with different color intensities according with the level of up-regulation or down-regulation). Blue bars denote the impact of each biological process.

**Fig 5:**
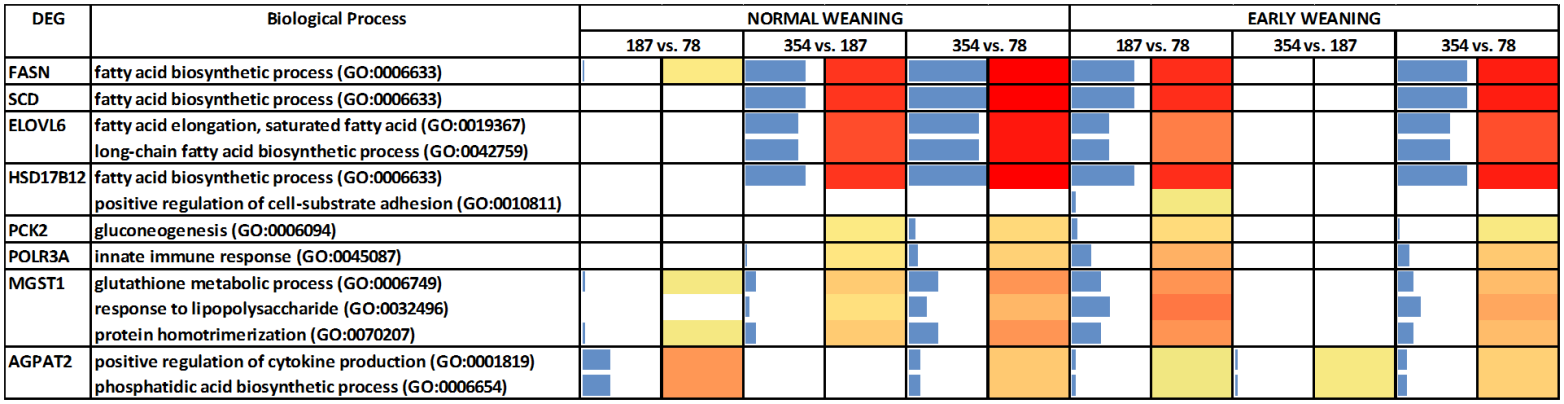
Significant biological processes (BP) among the differentially expressed genes reported in Table 3 for each time comparison. Flux represents the direction of each category and the corresponding subcategory (green color = inhibition, yellow color = stable, red color = activation with different color intensities according with the level of up-regulation or down-regulation). Blue bars denote the impact of each biological process.

**Fig 6:**
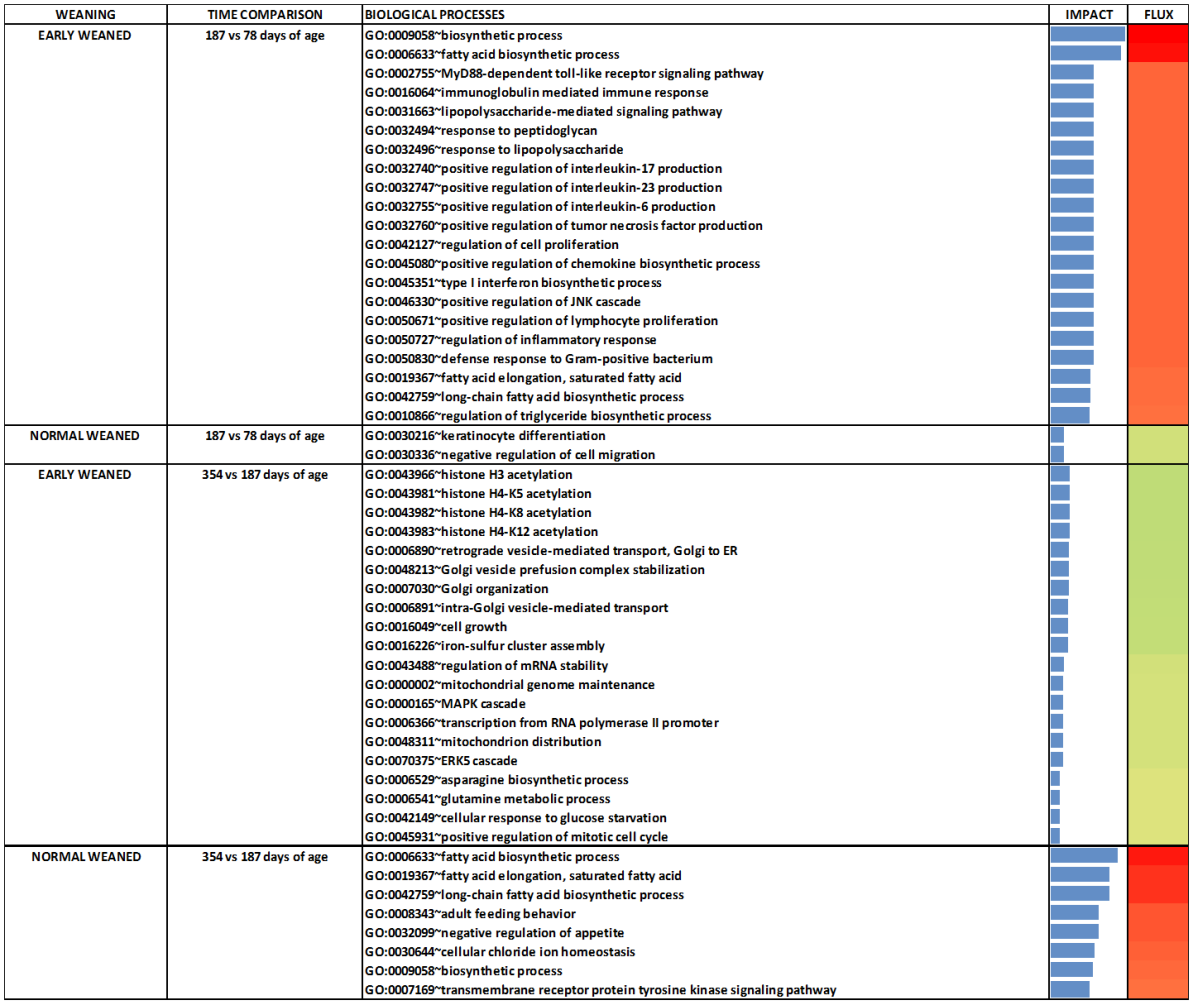
Significant biological processes in the comparison of differentially expressed genes between early wean (EW) and normal wean (NW) steers at 78 and 187 days of age and 187 and 354 days of age. Flux represents the direction of each category and the corresponding subcategory (green color = inhibition, yellow color = stable, red color = activation with different color intensities according with the level of up-regulation or down-regulation). Blue bars denote the impact of each biological process.

The DIA analysis was performed with DEG (n = 167) at an uncorrected *P* value = 0.05 and an FDR = 0.10. Fig 3 contains the summary of KEGG pathways results from DIA. The top-three most impacted canonical pathways are reported in Fig 4 and the genes with highest activation in Fig 5. For biological processes, only those with an impact value higher than 50% of the maximum total impact value for each time comparison for EW and NW steers are discussed (Fig 6).

The DIA analysis revealed that fatty acid biosynthetic process, biosynthesis of unsaturated fatty acids, and insulin signaling were highly-activated in EW steers between 78 and 187 days of age (Fig 4). Analyses further revealed that activation of these pathways was namely due to upregulation of *FASN* (lipogenic enzyme), *SCD* (monounsaturated fatty acid synthesis) and *PCK2* (involved in glyceroneogenesis) (Fig 5). In contrast to EW steers, between 78 and 187 days of age NW steers had a higher impact with no apparent direction of the cytochrome P450-related pathways and Glutathione Metabolism (Fig 4).

Between 187 and 354 days of age in EW steers there was a significant negative impact on Pyrimidine metabolism (Nucleotide Metabolism Subcategory within the Metabolism KEGG Category), RNA polymerase (Transcription Subcategory within the Genetic Information Processing KEGG Category) and Cytosolic DNA sensing pathway (Immune System Subcategory within the Organismal System KEGG Category) (Fig 4). Polymerase (RNA) III (DNA directed) polypeptide A, 155kDa (*POLR3A*) was the only DEG that could explain the inhibition of these pathways (Fig 5). In the case of NW steers between 187 and 354 days of age, Biosynthesis of unsaturated fatty acids, Fatty acid biosynthesis and Glycerophospholipid metabolism (all within the KEGG Lipid Metabolism Subcategory) were the most-impacted and activated pathways (Fig 4). Between 78 and 354 days of age, Fatty acid biosynthesis and Biosynthesis of unsaturated fatty acids were the most-impacted and activated pathways in both EW and NW, but the activation of these pathways took place at different time points (Fig 4).

When we focus on the biological processes (BP), between 78 and 187 days of age in EW steers there was a clear activation of the BP related to adipogenesis and lipogenesis (Fig 6). Biosynthetic process and fatty acid biosynthetic process had the highest impact, with a lower impact detected for fatty acid elongation of saturated fatty acids, long-chain fatty acid biosynthetic process and regulation of triglyceride biosynthetic process (Fig 6). Between 78 and 187 days of age in EW steers, most of the biological processes affected by weaning age that had an impact value higher than 50% of the maximum total impact were processes related to inflammation, innate immune response and lipogenesis. In contrast, in NW steers, only keratinocyte differentiation and negative regulation of cell migration had an impact level greater than 50% of the maximum (Fig 6).

Between 187 and 354 days of age, EW steers had an inhibition of histone H3, H4-K5, H4-K8 and H4-K12 acetylation, retrograde vesicle-mediated transport from Golgi to endoplasmic reticulum, Golgi vesicle prefusion complex stabilization and organization, intra-Golgi vesicle-mediated transport, cell growth, iron-sulfur cluster assembly, and other pathways that had a lower impact value (Fig 6).

## Discussion

### Animal performance and blood metabolites

As reported previously [13], the greater initial BW for EW steers was a result of feeding of the high-grain (“finishing”) diet at an earlier age. The NW-MPN steers gained more kg of BW at the feedlot compared with other treatments because they were less efficient in terms of fat accumulation, hence, remained longer at the feedlot until reaching back-fat target end-points. Although our data cannot provide a mechanistic explanation, the fact that LPN steers ate less than MPN steers underscores a strong impact of cow plane of nutrition (LPN or MPN) on the intake of EW steers.

In non-ruminants, adiponectin is involved in regulating systemic glucose levels as well as hepatic fatty acid oxidation [14]. In a previous study, a higher adiponectin concentration was associated with a decrease in insulin sensitivity in humans [15]. The minor changes in serum insulin and glucose concentrations between 78 and 187 days of age in all steers appear to indicate an effect of adiponectin on glucose homeostasis due to its increasing levels during that period of time. Higher plasma insulin concentrations between 187 and 296 days of age are common in high-starch fed animals, and coupled with the decrease in glucose concentration during the same time-frame it suggests greater glucose uptake from the bloodstream.

### Cow plane of nutrition, weaning age and time

The DEG affected by the interaction of cow plane of nutrition (diet), weaning age and time encompass various biological processes (Table 4). The down-regulation of *NME1* suggests a potential inhibition of dynamin-dependent fission of coated vesicles at the caveolae or lipid rafts present in adipocytes [16]. Similarly, the down-regulation of *MAPK4* due to time, diet and weaning suggests a negative effect on adipose proliferation and differentiation as reported in vitro [17]. There are some DEG for which a biological role in skeletal muscle has not, to our knowledge, been reported. Thus, further research will have to be conducted to uncover the relevance of *OR51F1*, *IMPG1*, *KIAA0232*, *KRTDAP* and *SLC25A5*.

The change in expression of several transcription factors could reflect an overall decrease in gene transcription in LM, the overall effect of which could not be discerned in the present study. For instance, *ARID1A* associates with *E2F4* and *E2F5* and contributes to down-regulation of target promoters [18]. Up-regulation of *ARID1A* in our study could reflect a blockade of transcription potentially leading to cell cycle arrest [19] in the LM. *PLAGL1* encodes a growth suppressor protein and it shares with p53 the ability to induce both apoptosis and cell cycle arrest [20]. Therefore, the up-regulation of *PLAGL1* could be taken as an indication of LM growth inhibition (Table 4).

The induction of *EPAS1* enhanced intracellular lipid droplet accumulation [21]. Thus, the down-regulation of *EPAS1* in the present study could have limited a pro-adipogenic response that would have increased intramuscular fat accumulation (i.e. marbling). Although it is well-known that myocyte differentiation no longer occurs postnatally, the down-regulation of *BTG1* in LM also could have been related with intramuscular fat deposition because a previous study reported that *BTG1* has a role in regulating adipose-derived stem cell differentiation to osteocytes and myocytes [22]. Overall, the responses observed for all transcription regulators indicated a state of inhibition of cell proliferation and differentiation. It remains to be determined if those responses have a biological effect in terms of LM growth.

### Cow plane of nutrition effect

#### Adipose tissue inflammation

The LM in LPN steers had signs of an inflammatory response due to the up-regulation of signal peptide, CUB domain, EGF-like 1 (*SCUBE1*) and caspase recruitment domain family, member 14 (*CARD14*). *CARD14* interacts with T- and B- cell receptor complexes present in lipid rafts [23] and it functions to assemble these complexes at the plasma membrane to transduce distinct upstream stimuli leading to the activation of *BCL10* and nuclear factor kB (*NF-kB*) [24]. The strong up-regulation of *SCUBE1* and *CARD14* in LPN steers as compared to MPN steers suggests a localized inflammatory response due to the low plane of nutrition in utero (Table 3). The trigger for such localized inflammatory response could be associated with expression of Dynein (*DYNLL1*). Formation of microtubule cytoskeleton is important for macrophage spreading; therefore, *DYNLL1* up-regulation in LPN steers might be one factor allowing macrophage infiltration in intramuscular fat of LM.

#### Macrophage infiltration

Cell migration is dependent on the continuous organization of the actin cytoskeleton, which is regulated by members of the small Rho GTPase family [25]. *ARAP1* and *IQGAP1[26]* encode domains for Arf guanosine triphosphatase-activating protein (GAP) and Rho GAP [27]. The Arf family of GTP-binding proteins are regulators of membrane trafficking and actin remodeling. The actin cytoskeleton is a key mediator of cell polarization and helps direct migration of macrophages and neutrophils into tissues [28]. Together, the down-regulation of *ARAP1*, *IQGAP1* and *ART3* (which modifies the function of proteins by the addition or removal of ADP-ribose to an arginine residue) suggests an inhibition of actin cytoskeleton remodeling due to impaired ADP-ribosylation. Such response could have been associated with an inhibition of LM remodeling by macrophage infiltration in LPN steers (Table 4).

Despite signs of impaired innate immune response in LPN steers, the up-regulation of *EHD4* suggests a potential activation of endocytosis, perhaps of apoptotic debris from adipocyte-infiltrated macrophages [29]. *EHD4* controls receptor recycling by regulating their transport, and it co-localizes with vesicular and tubular structures, implying roles in the internalization of receptors, cytoskeletal dynamics and their transport to early endosomes [30]. The biological role of *EHD4* and the endocytosis process in LM from growing steers is still unknown.

The activation of the innate immune response in EW steers between 78 and 187 days of age appeared largely due to macrophage infiltration after LM was challenged by a greater rate of lipogenesis [29]. One of the first symptoms of inflammation in adipose tissue is hypoxia (i.e. due to a high degree of cell proliferation, oxygen availability does not supply cell requirements), leading to deregulated production and secretion of adipocytokines [31]. Thus, at a certain point during the fattening period, hyperplastic fat depots and inflammation could potentially reduce the capacity of adipose tissue for lipid storage and secretion of adipokines [32].

The inhibition of the receptor for Fc fragment of IgA (*FCAR* or *CD89*), which is expressed on immune cells (i.e. macrophages), suggests an inhibition of the immune response in LPN steers compared with MPN. IgA binds to *FCAR* and forms a complex that initiates a downstream cascade of events that trigger a variety of inflammatory responses including phagocytosis, release of inflammatory cytokines, and oxidative burst [33]. The down-regulation of *KRT74* could have decreased the formation of keratinocyte-derived chemokines which are highly expressed in preadipocytes [34]. Although they do not have an effect on adipogenesis, they induce adipocyte expression of inflammatory factors such as *IL1*, *IL6*, and *TNF* [34].

Insulin insensitivity could be a consequence of oxidative stress. In healthy animals, long-chain fatty acid infusions cause an increase in oxidative stress and insulin resistance that is reversed by the administration of antioxidants such as glutathione [35]. Glutathione S-transferase, alpha 4 (*GSTA4*) is involved in detoxification and protection of cells against chemical and oxidative stress [36]. *GSTA4* catalyzes the detoxification of hydroxynonenal (HNE) and related lipid peroxides by conjugation to glutathione (GSH) [37]. The down-regulation of *GSTA4* in LPN compared with MPN steers suggests they were not undergoing an overt oxidative stress condition despite the predicted increase hypertrophy and hyperplasia of adipocytes in LM due to the higher energy intake.

#### Glucocorticoid receptors

Mitochondrial glucocorticoid receptor (GR) regulates some enzymes with specific roles in the oxidative phosphorylation pathway [38]. In the absence of glucocorticoids, the glucocorticoid receptor (GR) resides in the cytosol (inactive) complexed mainly with heat shock protein 90 (hsp90) and heat shock protein 70 (hsp70) [39]. The translocase of outer mitochondrial membrane 34 (*TOMM34*) has a tetratricopeptide repeat domain (TPR1) that specifically binds Hsp70. *TOMM34* represents a novel scaffolding co-chaperone of Hsp70 and Hsp90, which may facilitate Hsp70/Hsp90 cooperation during protein folding or to keep the complex in an unfolded mitochondrial protein import-compatible state [40]. The up-regulation of *TOMM34* in LPN compared with MPN steers might have enhanced the role of GR in maintaining normal mitochondrial functions (i.e. oxidative phosphorylation) even under oxidative stress conditions (Table 3).

#### Regulation of transcription

The overall process of gene transcription could have been affected by the cow plane of nutrition by means of changes in expression of 3 DEG: hepatic leukemia factor (*HLF*), nuclear receptor subfamily 2, group C, member 2 (*NR2C2*) and enhancer of polycomb homolog 1 (*EPC1*). The knockdown of *HLF* significantly reduced lipid content in Drosophila[41], thus, the up-regulation of *HLF* in LPN steers as compared to MPN steers could have been associated with differences in intramuscular fat deposition (Table 3). Because *NR2C2* can act as a negative regulator of the retinoid signaling pathway [42], the down-regulation of *NR2C2* in LPN steers as compared to MPN steers could have played a role in the overall control of intramuscular fat deposition (Table 3). *EPC1* is part of a core repressor complex, with *E2F6* and *DP1*. In proliferating cells, *EZH2* binds to *EPC1* [43]. *EZH2* has histone methyltransferase activity and it was proven to elicit a pro-adipogenic effect [44]. Overall, these data suggest that at the transcriptional level a low maternal plane of nutrition favored intramuscular fat development in the offspring (Table 3).

#### Lipid metabolism

Lipid metabolism was affected by cow plane of nutrition by means of the up-regulation in LPN steers of ATP citrate lyase (*ACLY*) and the slight down-regulation of 1-acylglycerol-3-phosphate O-acyltransferase 6 (*AGPAT6*). The former is a key link between the metabolism of carbohydrates and the production of fatty acids [45] The role of AGPTA6 is to esterify the acyl-group from acyl-ACP to the sn-1 position of glycerol-3-phosphate, thus, participating in triacylglycerol synthesis [46]. In ruminant adipose tissue, ACLY activation (together with NADP-malate dehydrogenase) provides the necessary NADPH for fatty acid production from lactate [45]. Taken together, fatty acid metabolism in LM of LPN steers seems to have been more active in terms of lipogenesis. It also is possible that down-regulation of *AGPAT6* is a factor precluding normal triacylglycerol synthesis in steers with sub-optimal nutrition in utero.

### Weaning age and time effect

In the present study, the activation of the fatty acid biosynthetic process soon after early weaning confirmed the anabolic effect of feeding a high-grain diet at a young age. In contrast, only after the NW steers joined the EW steers at the feedlot (around 187 days of age) their LM had an activation of these metabolic pathways (Fig 6). Thus, high-dietary grain at an early age is a consistent trigger of adipogenesis and lipogenesis.

#### Innate immune response

The transcriptome response between 78 and 187 days of age revealed a clear activation of the innate immune system within LM in EW compared with NW steers. It is noteworthy that none of the EW steers biopsied had to be treated for bovine respiratory disease as compared to the herd from where these biopsied steers were selected[8]. Therefore, the overall activation of the innate immune response in LM of EW steers might have been a normal response to the greater dietary energy, i.e. more nutrient availability to cells (Fig 6). Normal activation of the innate immune response is exerted when fat depots reach a plateau in which excessive hyperplasia and hypertrophy and diminished oxygen availability inhibit adipogenesis and increase lipolysis and fatty acid release [47].

Among the innate immune pathways affected, the activation of the MyD88-dependent toll-like receptor signaling pathway in EW steers between 78 and 187 days of age denotes an inflammatory response potentially elicited by circulating cytokines acting within LM in a MyD88-dependent manner [48]. There is previous evidence demonstrating that chronic feeding of high-starch diets could increase the circulating concentration of endotoxin/LPS which can then trigger a pro-inflammatory response by tissue-associated immune cells [49],[50]. Another sign of the activation of the innate immune response in EW steers was the activation of the JNK cascade which is an intermediary molecule in the cascade of events leading to the synthesis of cytokines. JNK is one of the four well-characterized subfamilies of MAP kinases (MAPKs) [51]. MAPKs phosphorylate transcription factors and other targets to regulate gene transcription and immune responses.

A pseudo-inflammatory state because of infiltration of tissues by immunoreactive cells such as macrophages, lymphocytes, eosinophils, and mast cells triggers the release of inflammatory mediators such as interleukins [52]. IL-6 production activates B cell differentiation into plasma cells that are responsible for antibody secretion. IL-17 maintains cytokine production and IL-23 has a role in the maintenance of IL-17 producing cells [53] (Fig 6). Taken together with the positive regulation of interleukin production (*IL6*, *IL17* and *IL23* specifically) in EW steers between 78 and 187 days of age, the present data suggest that activation of lymphocyte proliferation and positive regulation of cytokines and interleukins are normal mechanisms that contribute to adipose tissue expansion. The present study confirms the existence of an innate immune response in LM of EW steers after weaning likely due to increased intramuscular fat accumulation.

Microsomal glutathione S-transferase 1 (*MGST1*) transcription, similar to some glutathione peroxidases, also is susceptible to oxidative stress [54]. In the present study, *MGST1* appears particularly important in the LM response to weaning because the bioinformatics analysis indicates that it influences several pathways in NW steers between 78 and 187 days of age (Fig 5). Among these pathways, those related to glutathione metabolism and cytochrome P450 had a slight inhibition in NW steers (Figs 4 and 5). In contrast, it is plausible that activation of these pathways in EW steers was related to the initial stages of an inflammatory process due to the earlier deposition of fat during the growing phase [29].

#### Myogenesis and adipogenesis

Chloride intracellular channel 4 (*CLIC4*) downregulation likely was responsible for the inhibition in NW steers of keratinocyte differentiation and negative regulation of cell migration between 78 and 187 days of age (Fig 6). *CLIC4* is considered a growth inhibitory protein, which in vivo is located in the nucleus. By altering the Cl^-^ and pH of the nucleus *CLIC4* contributes to cell cycle arrest and the specific gene expression program associated with conversion of fibroblasts into myofibroblasts [55]. The fact that keratinocyte differentiation was the biological process with the highest impact in NW steers between 78 and 187 days of age supports a role for *CLIC4* in LM. However, because the LM biopsy likely contained a combination of myocytes and adipocytes also could mean that *CLIC4* is important in the overall process that inhibits cell differentiation that could control muscle and fat growth in young steers.

#### Epigenetic regulation of transcription

Contrary to the precocious response in EW steers between 78 and 187 days of age, the LM of NW steers had a marked activation of the lipogenic biological processes between 187 and 354 days of age (Fig 6). In fact, during the same time-frame EW steers appeared to experience a “slow-down” in the regulation of transcription through inhibition of histone acetylation, which is an epigenetic-regulated process [56]. In our study, EW steers had an inhibition of histone H3 and H4 acetylation. The inhibition of histone H4-K5 acetylation is catalyzed by the enzyme encoded by *KAT5* and the CBP/p300 protein [57]. Regulation of acetylation by CBP/p300 has been related to an autoimmune response [58] suggesting that in our study transcriptional inhibition might have been triggered by the activation of the innate immune response between 78 and 187 days of age in EW steers (Fig 6). Histone H4-K8 acetylation and histone H4-K12 acetylation also were inhibited in EW steers between 187 and 354 days of age.

#### Lipid droplet formation

Nascent lipid droplets are secreted and transported from the endoplasmic reticulum (ER) to the Golgi apparatus [59]. The transport of lipid droplets requires the localization and activity of proteins that create ER exit sites, coat proteins to collect cargo and to change membrane's shape into a transport container, and address tags (SNARE proteins) to target the vesicles specifically to the Golgi apparatus. ER export factors, SNAREs, and misfolded proteins used during the release of lipid droplets must be retrieved from the Golgi to the ER again [60]. If this process is impaired, Golgi vesicle pre-fusion complex stabilization occurs (Fig 6). Lipid droplet dynamics, trafficking and dispersion was demonstrated to be produced by homotypic fusion between lipid droplets using SNAREs [61]. The inhibition of translocation of resident proteins from the Golgi to the ER, and endosomal trafficking to the biosynthetic/secretory compartments (so called retrograde trafficking) [62] between 187 and 354 days of age in EW steers potentially led to a diminished intra-Golgi vesicle-mediated transport (Fig 6). If such events occurred it might have led to reduced lipid droplet formation, hence, lower marbling in LM of EW steers.

## Conclusions

Cow plane of nutrition during the last 105 days of gestation resulted in modest changes in the offspring longissimus muscle transcriptome. Among these genes the bioinformatics analysis revealed an inflammatory process in offspring born from cows with access to higher plane of nutrition. The greater intramuscular lipogenic response in those steers contributed to the effect. Irrespective of maternal nutrition, early weaning induced a robust activation of the lipogenic program accompanied by activation of the innate immune system. The latter also was related to the greater lipogenesis during the growing phase. Subsequently, during the finishing phase, alterations of genes in the Golgi complex organization suggest an inhibition of lipid droplet formation potentially diminishing intramuscular fat accumulation in the steers.

When only genes affected by weaning age are considered, the inflammatory response detected at the end of the growing phase in early-weaned steers was similar to that observed when evaluating DEG affected by cow plane of nutrition; in that case with signs of adipose tissue inflammation for both, MPN and LPN steers. These mechanisms were more pronounced in early-weaned steers from cows fed low plane of nutrition probably because of the lower intramuscular fat (marbling) scores. Overall, results from this work underscore the need to conduct further studies to understand better the functional outcome of the transcriptome changes. Additional knowledge on molecular and functional outcomes induced by the combination of prenatal and postnatal nutritional management would help produce more efficient beef cattle and higher quality beef.
